# Zoonotic infections in UK and Irish veterinary students: a cross-sectional survey

**DOI:** 10.1186/s12889-024-18777-3

**Published:** 2024-05-09

**Authors:** Tamzin Furtado, Lois Kennedy, Gina Pinchbeck, John S. P. Tulloch

**Affiliations:** 1 Department of Livestock and One Health, Institute of Infection, Veterinary, and Ecological Sciences, University of Liverpool, Liverpool, CH64 7TE UK; 2 School of Veterinary Science, Institute of Infection, Veterinary, and Ecological Sciences, University of Liverpool, Liverpool, CH64 7TE UK

**Keywords:** Zoonosis, Veterinary, Work-place infection

## Abstract

**Background:**

Zoonotic infections are a recognised risk for the veterinary community. Veterinary students are at risk, due to the range of activities they participate with on training coupled with their inexperience; yet the prevalence and severity of infections in veterinary students has been little studied. In this study, a survey explored zoonotic infections in UK and Irish veterinary students.

**Methods:**

A survey containing both open and closed questions, was distributed to undergraduate veterinary students at all veterinary schools in the UK and Republic of Ireland. Descriptive statistics, and univariable logistic regression were used to explore quantitative data; thematic analysis was used to explore qualitative data.

**Results:**

There were 467 responses, 31.5% (95% CI 27.3–35.9, *n* = 147) of those students reported having contracted at least one zoonotic infection during their studies. The most prevalent self-reported infections were cryptosporidiosis (15.2% of all respondents), dermatophytosis (5.6%), and other gastrointestinal infections assumed to be of zoonotic origin (4.5%). 7% of respondents reported having acquired a zoonosis within the last 12 months, 91% of these infections were acquired during farm placements. Thematic analysis (*n* = 34) showed that infection was an accepted risk, particularly on farm, and students were often reluctant to take time off their studies or placements as a result of infection. Reporting was very low, meaning universities would not have accurate figures on infection risk or particularly risky placement providers.

**Conclusions:**

Based on these survey results, veterinary students appear to be at increased risk of contracting zoonotic diseases, particularly on farm placements. Attitude and behaviour change at multiple levels is required to reduce the risk of infection to students and normalise reporting of illness.

## Background

Zoonoses are an acknowledged occupational hazard in the veterinary community [1–3]. The prevalence of confirmed zoonotic infections among veterinarians varies between countries; 8% in the United States of America (USA) [4], 15% in Finland [2], 17% in Canada [5], and 24% in the United Kingdom (UK) [6]. The risk of acquisition for veterinarians is almost three times higher than that of human medical general practitioners [7]. The causal pathogens vary between clinical speciality and country of practice, but many studies record the following diseases as the most prevalent in their respective veterinarian populations; campylobacteriosis, dermatophytosis, Q fever, Methicillin-resistant *Staphylococcus aureus* (MRSA), salmonellosis, brucellosis, and sarcoptic mange [4–6, 8]. For example, in Denmark 36% of livestock veterinarians are seropositive to *Coxiella brunetti* [9], whilst 10% of Finnish veterinarians are seropositive to Hepatitis E Virus [10]. The majority of zoonotic pathogens involved are associated with food-producing animals rather than companion animals or equids.

Despite relatively extensive knowledge about zoonoses and their associated risks in the veterinary profession, very little is known about them in the veterinary student population. A single study explored the prevalence of self-reported zoonoses and the seroprevalence of Q fever in Dutch veterinary students [11]. 20% of students self-reported having had a zoonotic infection with the most prevalent diseases being dermatophytosis (8.5%), ‘other fungal infections’ (5.5%), campylobacteriosis (1.5%), and salmonellosis (1.2%). Despite no students reporting a Q fever infection, 19% were seropositive. Risk factors for being seropositive included, year of study (higher years had higher odds), those who were on the ‘farm animal health direction’ of the course, and if they had reported being infected with other zoonoses.

In the UK and Republic of Ireland (RoI), veterinary students receive formal animal husbandry, zoonotic diseases, and biosecurity training to achieve the following Royal College of Veterinary Surgeons (RCVS) Day One competencies [12]:

*‘Recognise suspicious signs of possible notifiable, reportable and zoonotic diseases and take appropriate action, including notifying the relevant authorities.*

*Recommend and evaluate protocols for biosecurity, and apply principles of biosecurity correctly, including sterilisation of equipment and disinfection of clothing.*

*Promote the health and safety of people and the environment*.’

Students receive this training through didactic and practical teaching at their respective universities [13]. However, a large part of their training and exposure to animals is through Extra-Mural Studies (EMS), at the time of the study this comprised of 12 weeks pre-clinical and 26 weeks of clinical placements [14, 15]. EMS aims to provide students with exposure and practical experience of all the facets of veterinary work. Typically, pre-clinical EMS placements are based at farms, kennels, and stables, whilst clinical EMS is based at veterinary practices. Awareness of zoonotic risk is a compulsory element of the course, but as the most inexperienced members of the veterinary community, students may be at greatest risk of contracting disease. In addition, there are risk factors unique to students; students are exposed to a greater number of species, more varied activities (dissection, necropsy) and locations (different farms, practices). This wider variety and lack of experience may make veterinary students more susceptible to zoonotic diseases.

Whether the students’ formal training is sufficient is not conclusive; a review of the literature concerning student zoonoses concluded that more training in zoonoses and biosecurity beginning day-1 was advisable to protect students from acquiring infection [16]. Practicing veterinarians may also influence the students when they teach, whether in a university setting or on EMS, and nonchalant attitudes towards zoonotic disease may be passed on to budding veterinarians, despite formal public health education, as part of the ‘hidden curriculum’ [17].

There remain many unknowns in relation to veterinary students and zoonotic infections, including the prevalence and type of infection, and students’ behaviour and attitude to an infection post-acquisition. It is important that these knowledge gaps are addressed, in order for universities to fulfil their obligations in relation to student safety, to ensure that students are taught skills to mitigate against risk and to avoid adverse effects of zoonoses which could impact students’ ability and desire to participate in EMS placements and impact their choice of career path. Therefore, a closer understanding of students’ experiences of zoonotic infections, their attitudes to infection, and their knowledge of reporting structures are important areas for study.

This study aims to investigate the prevalence and attitudes towards zoonoses, consequences, and reporting culture in the veterinary student population.

## Methods

An online cross-sectional survey was designed, incorporating both closed and open questions in order to allow rich description if necessary. Respondents were initially asked about personal demographics (i.e. sex, age, nationality), details of their university, year of study, and stage of course (i.e. pre-clinical or clinical). Respondents were then asked about zoonoses they had acquired during their studies, and more detailed questions if they had acquired an infection in the last 12 months. This included questions on where the infection was acquired, potential species involved, medical consequence, student attitude to the infection, any resultant behaviour change, and the reporting process. The survey was piloted with a small group of veterinary students and then distributed through social media via accounts with large veterinary student followings, such as university veterinary societies. It was additionally distributed through official veterinary school emails. Any current veterinary student in the UK and RoI, studying at one of the ten veterinary schools, was eligible to participate in this survey. The survey was open from July 8th 2021 to August 31st 2021. Reminders were posted on social media weekly whilst the survey was open.

Analysis of results included the description of demographic characteristics. Overall zoonotic infection prevalence was calculated and stratified by demographic data. Logistic regression was performed to identify any association between demographic variables and zoonotic infection. Multicollinearity was checked between independent variables. If not correlated, variables taken forward for multivariable analysis were selected through substantive knowledge and significance (i.e. where p < = 0.3), with veterinary school treated as a fixed effect. Hosmer-Lemeshow tests were performed to assess goodness of fit. The mean number of reported zoonotic infections was calculated per student. The prevalence of named zoonotic pathogens acquired at any time during the veterinary degree, and during the twelve months prior to the survey (annual prevalence), was calculated. The animal species involved and location the zoonoses was acquired were reported descriptively.

Open text questions related to the context of infection, attitude of infected respondent, and reporting culture, and analysis of these items was carried out using an iterative thematic analysis [18]. Initially, the responses were read through with the researchers making note of any initial impressions; secondly, the researchers carried out initial “coding”, by reading items individually and labelling important concepts; for example, an initial code might relate to a “sense of personal responsibility” over avoiding infection. As more text was incorporated into the analysis, codes were refined, combined, or renamed to more accurately represent the information conveyed by respondents. Eventually, codes could be grouped into overall categories or “themes”.

## Results

There were 467 responses, 31.5% (95% CI 27.3–35.9, *n* = 147) of those students reported having contracted at least one zoonotic infection during their studies. The estimated veterinary student population in 2021 in the UK and RoI was 7241 [19–21]. If all students were exposed to the survey, then the crude response rate was 6.4%. The majority of students were; female, aged 18–24, white, and British (Table 1). Demographic representativeness was hard to establish as no publicly available information about student demography exists. However, we believe the participants to be broadly representative as 90% of the survey respondents were female compared to 81% in the practising population, and 96% of the veterinary profession identifies as white compared to 94% of the respondents [22]. Responses came from all ten veterinary schools, though number of responses by veterinary school was not proportionate to the size of the school. More students within the clinical stages of their degree responded. Univariable analysis revealed that there was no association between any demographic variable or attendance of any one university and the odds of acquiring a zoonotic infection (Table 1). The only significant associations were between year, and ‘stage of the degree’, and zoonoses acquisition. The longer a student had been on the course the greater the odds of acquiring a zoonoses, and students in their clinical years were more likely to have a acquired a zoonoses than if they were in the preclinical part of the course. Due to strong collinearity (*r* = 0.86) between the two univariable variables of interest (‘Year of Degree’ and ‘Stage of Degree’), a multivariable model was not created.

**Table 1:** Univariable and multivariable logistic regression (adjusted for all variables in the table) exploring demographic variables associated with acquiring a self-reported zoonotic infection in UK and Irish veterinary students

Demographic Variables	Respondent Characteristics (95% CI)	Zoonosis Prevalence (95% CI)	Univariable analysis	
			Odds ratio (95% CI)	*p*-value
**Sex**				
Female (ref)	90.3% (87.2–92.8)	31.9% (27.4–36.6)		
Male	9.7% (7.2–12.8)	31.1% (18.2–46.7)	0.96 (0.48–1.84)	0.92
**Age**				
18–24 (ref)	81.6% (77.8–85.0)	44.3% (38.2–50.5)		
25–29	16.1% (12.9–19.7)	36.0% (25.2–47.9)	1.27 (0.75–2.21)	0.37
30+	2.4% (1.2–4.2)	27.3 (6.0–61.0)	0.85 (0.18–2.98)	0.81
**Ethnicity**				
White (ref)	93.5% (90.9–95.6)	31.5% (27.2–36.1)		
All other ethnic groups combined	6.5% (4.4–9.1)	33.3% (17.3–52.8)	1.09 (0.48–2.34)	0.83
**Nationality**				
British (Ref)	76.9% (72.8–80.6)	29.5% (24.9–34.5)		
American	4.9% (3.1–7.3)	47.8% (26.8–69.4)	2.19 (0.92–5.14)	0.07
Canadian	2.6% (1.3–4.4)	25.0% (5.5–57.2)	0.80 (0.17–2.72)	0.74
Irish	9.2% (6.7–12.2)	32.6% (19.1–48.5)	1.15 (0.57–2.23)	0.68
All other nationalities combined	6.4% (4.4-9.0)	43.3% (25.5–62.6)	1.83 (0.84–3.88)	0.12
**Disabilities**				
Abled students (ref)	90.4% (87.3–92.9)	30.8% (26.4–35.5)		
Students with self-identified disabilities	9.6% (7.1–12.7)	37.8% (23.8–53.5)	1.36 (0.71–2.56)	0.34
**Institution**				
University of Liverpool (ref)	22.5% (18.8–26.6)	30.5% (21.9–40.2)		
Harper Adams University	1.3% (0.5–2.8)	33.3% (4.3–77.7)	1.14 (0.15–6.16)	0.88
Royal Veterinary College	10.5% (7.9–13.6)	32.6% (20.0-47.5)	1.11 (0.53–2.27)	0.79
University College Dublin	13.5% (10.5–16.9)	34.9% (23.3–48.0)	1.22 (0.63–2.37)	0.55
University of Bristol	15.8% (12.7–19.5)	29.7% (19.7–41.5)	0.97 (0.50–1.84)	0.91
University of Cambridge	5.8% (3.8–8.3)	29.6% (13.8–50.2)	0.96 (0.36–2.36)	0.93
University of Edinburgh	5.1% (3.3–7.6)	20.8% (7.1–42.2)	0.60 (0.19–1.65)	0.35
University of Glasgow	4.3% (2.6–6.5)	40.0% (19.1–64.0)	1.52 (0.55–4.04)	0.40
University of Nottingham	16.1% (12.9–19.7)	28.0% (18.2–39.6)	0.89 (0.46–1.70)	0.72
University of Surrey	5.1% (3.3–7.6)	45.8% (25.6–67.2)	1.93 (0.77–4.78)	0.15
**Year of Degree** (as a linear term)	NA	NA	1.20 (1.05–1.37)	< 0.001
**Stage of Degree**				
Pre-clinical (ref)	36.4% (32.0–41.0)	25.3% (19.0-32.5)		
Clinical	63.6% (59.1–68.0)	35.0% (29.6–40.7)	1.59 (1.05–2.44)	0.03

**Table 2:** The prevalence of self-reported zoonotic infections in UK and Irish veterinary students

Self-reported Zoonotic Infection	Prevalence of zoonoses acquired at any time during the veterinary degree (*n* = 467) (95% CI) NB some students acquired more than one zoonoses	Percentage of infections acquired in the last 12 months (*n* = 34) (95% CI)
No infection	68.5% (64.1–72.7)	NA
Cryptosporidiosis	15.2% (12.1–18.8)	35.3% (19.8–53.5)
Dermatophytosis	5.6% (3.7–8.1)	11.8% (3.3–27.5)
Gastrointestinal infections assumed to be of zoonotic origin	4.5% (2.8–6.8)	14.7% (5.0-31.1)
*E. coli* infection	1.9% (0.9–3.6)	5.9% (0.7–19.7)
*Orf virus* infection	1.9% (0.9–3.6)	14.7% (5.0-31.1)
Campylobacteriosis	1.3% (0.5–2.8)	2.9% (0.1–15.3)
Leptospirosis	0.6% (0.1–1.9)	2.9% (0.1–15.3)
Salmonellosis	0.6% (0.1–1.9)	2.9% (0.1–15.3)
Mite infestation (unknown species)	0.6% (0.1–1.9)	0
*Rotavirus* infection	0.4% (0.1–1.5)	5.9% (0.7–19.7)
Lyme disease	0.2% (0.0-1.2)	0
*Herpesvirus* infection	0.2% (0.0-1.2)	0
*Scabies* infestation	0.2% (0.0-1.2)	0
*Pseudocowpox* infection	0.2% (0.0-1.2)	0
Psittacosis	0.2% (0.0-1.2)	0
Q-fever	0.2% (0.0-1.2)	0
Worms (unknown species)	0.2% (0.0-1.2)	0
Unknown skin infection	0.2% (0.0-1.2)	2.9% (0.1–15.3)

The mean number of zoonoses students acquired during their studies was 1, with 85.2% (95% CI 78.3–90.6%) of respondents selecting this option, 12.7% (95% CI 7.7–19.3) of students acquired two zoonoses. The most prevalent self-reported infections were cryptosporidiosis (15.2% of all respondents), dermatophytosis (5.6%), and gastrointestinal infections assumed to be of zoonotic origin (4.5%) (Table 2). Seven percent of respondents (7.3%, 95% CI 5.1–10.0, *n* = 34) had acquired a zoonosis within the last 12 months. Thirty-five percent of these were reported to be cryptosporidiosis, 14.7% orf infection, and 14.7% gastrointestinal infections assumed to be of zoonotic origin.

The remainder of the survey focused on the 34 respondents who had acquired a zoonoses in the last 12 months. It was reported that sheep were responsible for 56.3% (95% CI 37.7–73.6%) of zoonotic infections, followed by cattle 34.4% (95% CI 18.6–53.2%), cats 6.3% (95% CI 0.8–20.8%), and pigs 3.1% (95% CI 0.1–16.2). All infections were acquired in the UK and RoI. Ninety-one percent of infections were acquired on farms during EMS placements (90.6%, 95% CI 75.0–98.0), 20.7% of these were on pre-clinical EMS, whilst 79.3% were on clinical EMS. Of the remaining infections 6.3% were acquired (95% CI 0.8–20.8%) on university farms, and 3.1% (95% CI 0.1–16.2) at small animal practices. Many students (46.7%, 95% CI 28.3–65.7) perceived that their illness had arisen from a direct result of interacting with an animal known to have a zoonotic infection or as a result of an ongoing outbreak on the farm. Thematic analysis also reflected that students viewed these known outbreaks or sick animals as the most likely route of transmission:

‘*I was doing a rotation at the university dairy unit which involved disbudding calves known to have cryptosporidium. Around 7 days later I had severe stomach cramps, watery diarrhoea and vomiting.*’

However, their responses also indicated that they implicitly viewed farms as locations where zoonoses might occur, even without a known outbreak or sick animal:

‘*I was on my 2 week pre-clinical dairy placement and the day after I had finished I had major abdominal pains and sickness.*’

As a result, students described an awareness of the necessity for routine use of personal protective equipment (PPE) and hygiene facilities (such as handwashing stations) in order to protect against transmission, yet access to these was not always available. Ten percent (10.0%, 95% CI 2.1–26.5) mention that they were not provided with appropriate PPE on the placement, as further described in the following example:

‘*I was on a lambing placement, the farm had very poor hygiene practices, I had no access to gloves nor access to anywhere I could wash my hands near the lambing sheds.*’

Two students mentioned going to a primary care physician to receive a diagnosis and treatment. Thirty-seven percent of students (36.7%, 95% CI 19.9–56.1) stated that someone else on the premises had also fallen ill. Ten percent (10.0%, 95% CI 2.1–26.5) took less than a week to recover, 43.3% (95% CI 25.5–62.6) took one to two weeks, 36.6% (95%CI 19.9–56.1) took two to four weeks, and 10.0% (95%CI 2.1–26.5) took more than a month. Despite this only 23.3% (95%CI 9.9–42.3) took time off from their studies, of these, two students took less than one week off, 4 took one to two weeks off, and one took more than three weeks off. Of those that did not take time off, 60.9% (95% CI 38.5–80.3) described acquiring the infection outside of term time and so had time to recover around EMS placements or in holiday time:

‘*It was during the summer break. I did have to take time off of the placement but I made up for this once I had recovered.*’

Twenty-six percent (26.1%, 95% CI 10.2–48.4) stated that they were not ill enough to take time off work. 9% (8.7%, 95% CI 1.1–28.0) stated that they wanted to ‘tough it out’ and not let their EMS placement down:

‘*I knew the farmer needed help, so I just toughed it out to finish lambing.*’

Four percent (4.3%, 95% CI 0.1–22.0) did not want to lose out on their EMS experience so continued despite being ill:

‘*Didn’t want to miss the experience of lambing.*’

The majority of students (86.7%, 95% CI 69.3–96.2) reported no emotional or mental health impacts resultant of the infection. Seven percent (6.7%, 95% CI 0.8–22.1) reported feeling more anxious about farm-based EMS and zoonotic infections. One student described an increase in self-consciousness due to physical scarring, and one reported increased stress due to having to re-organise an EMS placement.

In terms of behaviour change as a result of the infection, responses varied considerably. Twenty-three percent (23.3%, 95% CI 9.9–42.3) of students would not change their behaviour in similar scenarios in the future, predominantly because they felt that they had already taken reasonably precautions to protect against disease, for example one student states:

‘*I did everything right on EMS so (I) have nothing I could really change.*’

This suggests that, to some degree, disease transmission is considered simply a routine risk of placements which cannot be mitigated against. However, more than half of students (53.3%, 95% CI 34.3–71.7) would improve hand washing and disinfectant more frequently, whilst 26.7% (95% CI 12.3–45.9) would now always wear PPE whilst working with animals:

‘*Wearing gloves and washing hands more frequently.*’

Two students placed the blame of infection on their universities, whom they felt had not given them enough information about zoonotic infections and PPE:

‘*I feel as though we are not given enough support/PPE to protect ourselves on placements (esp pre-clinical) from infection or the behaviour of people on placement*.’

Notably, some students also described taking additional personal responsibility for bringing their own PPE or handwashing equipment in order to reduce risk, suggesting that they do not feel they can rely on universities or placement providers to have appropriate resources in place:

‘*I made sure to wash my hands even more frequently than I already was and would in the future make sure that my clothes were washed more. Would also take my own gloves.*’

‘*I wash my hands more often. Bring disinfectants, also I will also start wearing a face mask to the farm I think….*’

Two students described that they would now avoid working with the animals that transmitted the infection:

*‘I avoid working with cows*.’

Overall, 30.4% (95% CI 26.3–34.8) of survey respondents did not know the zoonosis reporting procedure at their university. Eighty percent (80.0%, 95% CI 61.4–92.3) of those infected in the previous year did not report their infection to their university. Over half of these students did not know if they were required to report it (54.2%, 95% CI 32.8–74.5), and 29.2% (95% CI 12.6–51.1) felt that their infection was not severe enough to be reported. One student described being concerned about academic repercussions if they did not finish their placement, and one student felt guilty about reporting on the basis of the farmer’s comments:

*‘The farm owners told me “there are no bugs in the shed” so I felt bad*.’

In only one circumstances were actions by the university taken post-infection, where the student was excused from their university laboratory practicals.

## Discussion

This is the largest survey to date exploring zoonotic infections in veterinary students; almost a third of students self-reported at least one suspected zoonotic infection during their studies, higher than the 24% reported in the practising population. The findings are concerning, particularly around farm EMS placements, with students appearing to assume that becoming sick is “part and parcel” of placements. Additionally, students were not confident in the provision of PPE or handwashing stations on placements, or the provisions available from universities, and as a result they perceived the need to bring their own equipment. Protecting students from zoonoses requires behaviour change from universities, placement providers, and students themselves in order to normalise hygiene practices, risk mitigation practices and disease reporting. This study highlights issues at all levels which are potentially leaving students open to risk, impacting students’ studies, their willingness to spend time with animals of perceived high risk, and increasing the spread of disease to humans or animals.

This study found that students were increasingly likely to have experienced a zoonotic disease as they progressed through their degree, which is expected due to the cumulative time spent with animals during their studies. However, the highest was on-farm placements accounting for 90% of infections. The most prevalent self-reported infections (cryptosporidium and dermatophytosis) reflect this, and are representative of the prevalence of zoonotic infections seen in the British veterinary profession [6]. Students frequently described perceiving that they had acquired a zoonoses during farm placements, and some described preferring to avoid farm animals as a result, which could have concerning repercussions for the recruitment of farm veterinarians. Students were aware of a diseased animal being present only 47% of the time, and concerningly no PPE was available for 10% of students. Collectively, this suggests that preclinical students on-farm may require additional support and PPE, but also that hygiene may be poor on many farms. These farms should be encouraged to improve hygiene practices in order to protect human health. Particularly concerning was that students’ reporting of zoonoses was low, suggesting that farms with particularly poor practices would remain unidentifiable even if students were repeatedly infected on their premises.

Students’ attitudes and experiences are synonymous with veterinarians’ attitudes to zoonoses in other countries. They were unaware of appropriate PPE usage or how to perform infection control practices that would reduce zoonotic disease transmission [23]. For example, a UK study of veterinarians found that PPE is under-utilised in practice due to time and safety concerns, as well as a feeling that clients and other professionals might perceive the individual to be over-cautious [6]. As a result, 18% of veterinarians described “hoping for the best” in relation to avoiding zoonoses, with less time in practice related to a more fatalistic attitude to infection. Similarly, a study of Finnish veterinarians found that while small animal vets’ glove-use was high (85%), on farm, glove-use was much lower (62%) [2]. In the USA, PPE was available only 69% of the time for livestock veterinarians, and noted barriers for PPE use were their apparent inconvenience, and reduced mobility whilst wearing them [4]. Combined with the results of the present study, these findings indicate that increasing availability of PPE and working to change social norms around PPE use, particularly on farm, could be key to altering attitudes and behaviour in student veterinarians and qualified veterinarians alike.

Students described a willingness to take more precautions after having become infected with a zoonotic disease; for example, they described a renewed understanding of the need to engage in frequent handwashing or changing clothes more frequently. This mirrors data in other sectors, in which personal experience of an illness can contribute to a “pivotal moment”; an event which leads to motivation for behaviour change [24]. However, avoiding becoming infected with zoonoses in advance would be preferable, and it was concerning that the on-farm and veterinary culture found in this study and others [2, 6, 23] suggest a culture of bravado and being “tough” in not using PPE, and not reporting their illness. This finding reflects broader cultural norms around work-based animal handling, where the safety of the animal is often prioritised over the safety of the human, and bravado is a cultural norm. For example, in the equine field where head injuries are commonplace [25], veterinary professionals are encouraged to wear helmets, yet rarely do so due to social norms and the desire not to show weakness or fear [26, 27]. In human medicine progress has been made in relation to clinical behaviours such as PPE use by changing social norms, and highlighting this change so that others are encouraged to comply with the new norm [28, 29]. Working to change social norms in the veterinary profession around zoonotic disease avoidance and reporting could therefore be a useful avenue for creating meaningful change.

There was discordance in the degree of sickness and the time that students took off from their studies. Eighty percent of students were sick for between one to four weeks, yet only 23% took time off from their studies. This is similar to practising veterinarians where 11% needed medical treatment, yet only 4% missed more than one day of work [5]. Overwhelmingly, students felt that they had no time to recover around EMS placements, which may have been partly a result of the pressures to gain a certain number of weeks’ experience. However, it was also clear that a sense of bravado was perceived as necessary and normalised; some students did not take time off or inform their university of their illness because they did not want to appear weak, let their placement down or lose out on their learning experience. Additionally, mechanisms for reporting sickness to universities were little-known and under-used, suggesting that universities will not have accurate figures on the number and severity of infections and illnesses. These findings are echoed in human medical practice, in which doctors are much less likely than the general population to take sick leave, citing concerns over the workload of colleagues and pressure on consultants [30–32]. As well as attending work when sick, other “maladaptive” coping strategies that doctors utilise are self-management of illness and self-prescribing medications. Reversing the normalisation of such practice is seen as key in human medicine, and our results suggest that a similar approach could be required in veterinary medicine.

The main limitation of this study was sample size. Despite many respondents experiencing zoonotic infections, only a minority had experienced a zoonoses in the previous twelve months. We had anticipated a higher proportion and as such we had limited responses to the more detailed section of the survey. This is likely due to the study period being during the first 18 months of the COVID-19 pandemic. At this time students were participating in fewer EMS placements due to national public health restrictions, which resulted in less contact time with animals, potentially lowering the risk of a zoonotic infection. This survey likely underestimates the prevalence of zoonotic disease in a non-pandemic affected veterinary student population. This survey relies on self-reported cases, which in most instances are unlikely to have been diagnosed by a medical professional. Out of 34 respondents detailing a zoonoses in the previous twelve months only 2 sought medical treatment and diagnosis. There is the possibility of misdiagnoses, many of the students mentioned gastro-intestinal illnesses and assume they have been caused by a zoonotic agent, whilst they could have been caused by several different pathogens. Additionally, some zoonoses have long incubation periods (i.e. tuberculosis) or have sub-clinical infections (i.e. toxoplasmosis), and may not have been recognised by the students, nor their symptoms presented. Most conditions described by the students have short incubation periods with clear symptomatology. As such, this work is likely to underestimate reported prevalence of some zoonotic infections. Thus, cases may have been over-reported, but conversely some students may have had a zoonotic infection, not received a diagnosis, and not reported it in this survey. The degree of under or over-reporting remains unknown and without all suspect cases being tested, or a seroprevalence study, it will remain challenging to get a highly accurate estimate of specific zoonotic disease prevalence. The crude response rate was low, however the survey was primarily disseminated through social media, with associated selection biases, and we do not know the number of veterinary students that were actually exposed to the survey. Veterinary students may have a degree of survey fatigue as they are sent multiple course assessment surveys each semester as part of their degrees [33, 34]. Survey fatigue was heightened during the COVID-19 pandemic when this study took place [34]. The lack of incentive, and the survey topic, may additionally have decreased response rates [33]. Self-selection bias may have been present, as students who felt that they had contracted a zoonotic infection could have been more inclined to participate. However, the demographics of the students appear to be representative and as such feel the potential for bias here to be small.

## Conclusions

This study provides, to our knowledge, the most in-depth study to date of student zoonotic infection. Both the high prevalence of infection and the findings around prevention, management, and reporting of zoonoses are concerning, particularly in relation to infections gained on farm placements. We identify farm placements as the highest risk for zoonotic disease transmission, and as placements where students are frequently exposed to situations where they do not have adequate PPE or handwashing facilities. Concerningly, the experiences on such placements were reportedly causing some students to dislike farm placements, or working with farm animals; this has repercussions for the future of the farm veterinary industry. These placements could be targeted for intervention to decrease the likelihood of ongoing transmission.

The concerningly low reporting of zoonotic infections represents a public health risk and suggests that either universities do not have clear reporting guidelines or that students are unaware of them. Consequently, they have a limited ability to monitor student health, and cannot identify “repeat offenders” or particularly risky placements. Additionally, the bravado described by students in attending workplaces while sick, or not taking time away from work or studies, represents a concern for the social norms around workplace sickness in the veterinary industry as a whole.

This project highlights the importance of encouraging attitude and behaviour change at multiple levels. These include universities improving their mechanisms for identifying and recording illnesses; students having an improved awareness of disease risk and reporting of illness; and placement providers being encouraged to increase hygiene practices and provisions for students.

## Data Availability

The datasets used and analysed during the current study are available from the corresponding author on reasonable request.

## Abbreviations

CI: Confidence Interval
EMS: Extra-Mural Studies
MRSA: Methicillin-resistant *Staphylococcus aureus*
PPE: Personal protective equipment
RoI: Republic of Ireland
RCVS: Royal College of Veterinary Surgeons
UK: United Kingdom
USA: United States of America
